# The ambulatory battery of creativity: Additional evidence for reliability and validity

**DOI:** 10.3389/fpsyg.2022.964206

**Published:** 2022-09-16

**Authors:** Christian Rominger, Andreas Fink, Mathias Benedek, Bernhard Weber, Corinna M. Perchtold-Stefan, Andreas R. Schwerdtfeger

**Affiliations:** Department of Psychology, University of Graz, Graz, Austria

**Keywords:** creativity, ecological momentary assessment, ecological validity, smartphone, mobile phone, experience sampling

## Abstract

Psychometrically sound instruments that assess temporal dynamics of creative abilities are limited. The Ambulatory Battery of Creativity (ABC) is designed to assess creative ideation performance multiple times in everyday life and was proven to capture the intra-individual dynamic of creative abilities reliably and validly. The present ambulatory study aimed to replicate and extend the psychometric evidence of the novel ABC. Sixty-nine participants worked on the ABC during a 5-day ambulatory assessment protocol. Each day, participants completed six randomly presented items of the verbal and the figural ABC. Matching previous psychometric analyses, the results indicated good between-person (≥0.80) and good within-person (≥0.72) reliability. Furthermore, evidence for between-person and within-person validity of the ABC was obtained. Performance in the verbal and the figural ABC were interrelated and correlated with an independent measure of creative potential. The verbal ABC was further associated with openness, self-reported creative behavior, creative activities, and creative achievements, thus providing additional evidence of construct validity, especially for the verbal ABC. Finally, the verbal and the figural ABC yielded convincing within-person validity: Longer response times and higher subjective originality ratings were associated with more original ideas. This replication and extension of the ABC’s psychometric properties indicates that it enables a reliable and valid assessment of moment-to-moment fluctuations of creative ideation abilities in everyday life, which may facilitate the investigation of exciting new research questions related to dynamic aspects of creative ability.

## Introduction

Creative potential is the skill of a person to produce novel and useful ideas (Dietrich, 2004; Runco and Jaeger, 2012; Benedek et al., 2014). Creativity research typically aims to assess participants’ creative potential during a single appointment, mostly in the laboratories, and sometimes in less controlled settings. This approach aims to control for situational and contextual influences by keeping them as stable as possible. The reduction of situational (error-) variance is thought to increase the measurement accuracy of people’s creative abilities (Guilford, 1950). However, this valuable and fruitful approach has one prominent pitfall. It misses the chance to assess the temporal dynamics and within-person variation of creative ideation abilities, which constitute important and meaningful aspects of creativity (Barbot, in press; Corazza et al., in press; Rominger et al., in pressa). In line with this, neuroscientific research indicated that meaningful within-person brain dynamics are linked with creative ideation performance (Schwab et al., 2014; Beaty et al., 2016; Rominger et al., 2019b,2020b,in pressc; Agnoli et al., 2020). However, the comparatively low number of studies on intra-individual dynamics of creative abilities might be due to the absence of proper psychometric methods, which would allow a reliable assessment of meaningful fluctuations in creative ideation performance (but see e.g., serial order effect in idea generation; Beaty and Silvia, 2012). This seems even more pronounced, when creativity researchers target to assess creative ideation performance in people’s everyday life (Rominger et al., in pressa).

The Ambulatory Battery of Creativity (ABC) measures creative abilities and their fluctuations in everyday life contexts by a repeated application of divergent thinking task items (Rominger et al., in pressb). The battery consists of a verbal and a figural version embedded in a signal-contingent ecological momentary assessment protocol (Shiffman et al., 2008). The ABC runs on smartphones, which prompt participants to find the most original use for an everyday object or the most original completion of a picture fragment. In contrast to more conventional assessment data, ecological momentary assessment data capture ecologically valid and meaningful within-person variation (covariations across measurements) and additionally allow to assess reliable between-person variance (aggregation across measurement prompts and items; Shrout and Lane, 2012; Bolger and Laurenceau, 2013; Nezlek, 2017; Sliwinski et al., 2018). The assessment of creative abilities in continuously changing situations in a natural environment allows to estimate between- and within-person variance, as well as the reliability indices by applying the generalizability theory analysis (Cronbach, 1972; Brennan, 2001, for review see, Shrout and Lane, 2012).

A first application of the ABC in combination with the generalizability theory analysis indicated that 8% of the assessed creative ability variance was due to between-person and 33% was due to performance variation within a person (Rominger et al., in pressb). In contrast, Silvia et al. (2008) reported a between-person variance of 63% for a verbal divergent thinking task. This divergence of observed variance proportions between data of the ABC and a single assessment of creative abilities suggests that creative ideation performance shows a high within-person fluctuation, which can only be observed when we assess creative ideation performance multiple times throughout various situations (Rominger et al., in pressb). On a theoretical basis, suggested that high within-person variations in creative ideation performance can be assumed, since numerous contextual variables such as affect (Baas et al., 2008; De Dreu et al., 2008; Nijstad et al., 2010), physical activity (Rominger et al., 2022), heart rate variability (Silvia et al., 2014; Rominger et al., 2019a), substance intake (e.g., alcohol, Benedek et al., 2017; caffeine, Zabelina and Silvia, 2020), semantic context (Fink et al., 2012), basic cognitive functions (e.g., executive functions, Zabelina et al., 2019; memory, Benedek et al., 2018; attention, Benedek, 2018) affect creative ideation performance and all these variables continuously fluctuate as we live our daily lives. In line with this argumentation, the performance measure of basic cognitive functions such as working memory, assessed by means of an ambulatory version of the classical n-back task, showed comparable within-person variance proportion (Dirk and Schmiedek, 2016; Sliwinski et al., 2018). A replication of these distributions of variances would further justify the application of the ABC to capture creative abilities in real-life settings, which allows a reliable and meaningful differentiation between within- and between-person variation of creative abilities.

The study of Rominger et al. (in pressb) indicated good within-person reliability of 0.70, which was higher than reported for basic cognition performance measures in the field (e.g., Sliwinski et al., 2018; see also Schmiedek et al., 2013; Brose et al., 2014; Schuster et al., 2015; Calamia, 2019; for an overview see Moore et al., 2017). In analogy to the convincing within-person reliably, the between-person scores showed good reliability and stability indices as well (≥0.80; for other cognitive tests see e.g., van Patten et al., 2021). To sum up, Rominger et al. (in pressb) indicated that the ABC can reliably assess within- and between-person variance in creative abilities. The promising reliability of the ABC argues for the assessment of creative ideation performance in everyday life situations, and the application in future studies (Rominger et al., in pressa). Importantly, the findings are not restricted to good reliability estimates, since the ABC additionally shows evidence of criterion validity.

Specifically, the within-person validity was indicated by a significant prediction of creative abilities via the response time and the subjective originality rating of each single prompt. Prompts with longer response times and higher subjective ratings were associated with a better performance in the verbal and figural ABC (Rominger et al., in pressb). First, this pattern of findings strengthens the assumption that response time can serve as an index of creative exploration (Barbot, 2018; Rominger et al., in pressb). In line with the serial order effect creative ideas seemed to increase across time (i.e., Beaty and Silvia, 2012). Second, the findings are in accordance with the view that people can monitor and evaluate their own ideas (i.e., creative metacognition; Karwowski et al., 2020). Furthermore, a positive association of the between-person score with self-rated creative behavior and the personality trait openness (McCrae, 1987; Silvia, 2008) add to the validity evidence of the verbal ABC. The remaining four Big-Five factors were not significantly linked to the ABC performance. This pattern of finding is in some agreement with literature indicating openness and extraversion as the two most important predictors of creativity (Puryear et al., 2017). The figural ABC performance was significantly associated with the verbal ABC performance. Although the study of Rominger et al. (in pressb) offered convincing first evidence for validity, associations with real-life creativity have not been explored to date. Kaufman and Beghetto (2009) discriminated between four types of creativity: Mini-c, Little-c, Pro-c, and Big-c. While Mini-c refers to personally meaningful creative experiences, Little-c refers to everyday creativity and creative activities. Pro-c is not as eminent as Big-c, however, it receives some public approval such as publishing a paper or blogging recipes. Thus, to assess everyday creative activities (capturing aspects of Little-c) and creative achievements (capturing aspects of Pro-c), we administered the Inventory of Creative Activities and Achievements (ICAA), developed by Diedrich et al. (2018). Both scales of the ICAA were previously associated with the performance in Alternate Uses tasks (AU-task; Guilford, 1967; see Diedrich et al., 2018).

Taken together, the present study aimed (1) to replicate the findings by Rominger et al. (in pressb). First, we were interested if the within-person variation of creative ideation performance would be comparably high again (compared to between-person variation) and second, we investigated if the ABC assesses between- and within-person creative ideation performance reliably and validly (by means of the very same approach used by Rominger et al., in pressb). (2) We aimed to extend the criterion validity of the ABC to real-life creativity assessments by applying the ICAA (Jauk et al., 2014; Diedrich et al., 2018). (3) We investigated additional indications of within-person validity of the assessed fluctuations of creative abilities by taking contextual factors into account. In particular we assessed the consumption of alcohol, caffeine, and nicotine, which were found to impact creative idea generation performance in experimental settings (Benedek et al., 2017; Zabelina and Silvia, 2020).

## Materials and methods

### Participants

A total of 69 students participated in this study. An a-priori calculated power analysis using the software GPower 3.1 (Faul et al., 2009) indicated that a sample size of 59 participants was required to detect a medium to large effect (*r* = 0.35) for validation analyses of between-person variance. The sample showed an age range between 18 and 54 years (*M* = 23.78, *SD* = 5.06; 45 women). Forty-five participants were majoring in psychology (65.20%). All participants were free of cardiovascular, neurological, or mental disorders as well as psychotropic or cardiovascular medication according to self-report. Participants were recruited via email and social media. Depending on the number of answered prompts, participants received between 15 and 30 Euros for participation (Gerteis and Schwerdtfeger, 2016). The study was approved by the institutional ethics review board (GZ. 39/100/63 ex 2020/21). All participants gave informed consent to participate in the study. No participant was excluded from analyses.

### Procedure

The study included one online meeting and two appointments (one to distribute and one to collect the equipment). During an online meeting, participants gave informed consent and worked on all relevant questionnaires delivered as an online survey. On the following day, at the first appointment, participants received detailed information on how to install the required software and to answer the prompts of the ABC. A short user manual was distributed including information on app use, frequently asked questions, time schedule of the study, and staff contact information. Participants were monitored throughout five consecutive days including weekends. A minimum of 4 prompts and a maximum of 10 prompts were delivered each day between 9:00 a.m. and 10:30 p.m. for each version of the ABC (verbal and figural). During ambulatory assessment, participants could contact the experimenter via email and the chat function implemented in the movisensXS app. After ambulatory assessment, participants returned the equipment (e.g., smartphones provided for non-Android users) and received their remuneration.

### Material

#### Ambulatory battery of creativity

We collected creative ideation performance data in the verbal and figural domain in everyday life via the Android-based app movisensXS (Version 1.4.3, movisens GmbH). Acoustic signals reminded participants to answer the prompts, which could be declined or delayed for either 5-, 10-, 15-, or 20-min. Multiple delays could not exceed 20 min in total.

##### Verbal ambulatory battery of creativity

The verbal ABC is a modified version of the Alternative Uses task (AU-task; Guilford, 1967). Thirty conventional everyday objects are randomly applied (see e.g., Fink et al., 2007, 2009). Within a time-limit of 60 s, participants generate one best possible creative and original use for an object. The response time from item onset to the start of typing the idea into the smartphone served as an index of creative exploration (and creative ideation; Rominger et al., in pressb; *M* = 14.27 s, *SD* = 13.83 s). Finally, participants rated the originality of their produced idea on a visual analog scale from “not original at all” to “very original” (ranging from 0 to 100, *M* = 46.26, *SD* = 23.09; Rominger et al., 2019b; Rominger et al., in pressb).

##### Figural ambulatory battery of creativity

Creative ideation performance in the figural domain was assessed by means of a modified version of the Picture completion task (Torrance Test of Creative Thinking, TTCT; Torrance, 1966). Participants were requested to complete picture fragments in a creative and original way on the display of their smartphone. The maximum time was again 60 s. Finally, participants were requested to give their drawings a title. The response time from item onset to starting the drawing was considered as an index of the exploration and creative ideation time in the creative thinking process (*M* = 10.53, *SD* = 7.89; Rominger et al., in pressb; Barbot, 2018; for a neurophysiological differentiation of idea generation and elaboration see e.g., Rominger et al., 2018, 2020b). The total drawing time (time until saving minus starting time) served as an index of creative idea elaboration (*M* = 27.79, *SD* = 15.09). In total, 30 picture fragments were randomly presented (20 from the picture completion task of the TTCT and 10 from the study of Rominger et al., 2018, 2020b). After completion, participants rated the originality of their drawing on an analogous scale from “not original at all” to “very original” ranging from 0 to 100, *M* = 48.83, *SD* = 24.15).

#### External originality ratings: Creative ideation performance in the verbal and figural version of the ambulatory battery of creativity

Four independent judges (two women and two men between 20 and 30 years of age) rated the creativity of all given responses (Silvia et al., 2008; Benedek et al., 2013; Forthmann et al., 2020). The judges had no extensive previous experience in originality ratings and were all well instructed. All raters were recruited via personal contact. The rating procedure was identical as described in Rominger et al. (in pressb). Similar ideas were removed (per item) to reduce the burden for judges, before the ideas (per item) were presented in random order to judges. The well instructed judges should gain an overview of all answers before providing the rating and should use the full range of the rating scale from 1 to 4 (not original to very original). In an analogous manner, all drawings (separately per picture fragment) were presented in random order to the same four judges. The figural ideas were rated by applying a rating-software (programmed in PsycoPy, Peirce, 2007), which randomly presented 10 completed drawings of the sample to the judges to gain an overview of participants’ drawings. After this, the judges rated one randomly presented drawing after the other with self-paced breaks between items. Each judge did the ratings at home and in isolation. The ratings took between 3 days and 1 week in total. The inter-rater-reliability (two-way random effects, consistency, multiple raters) as measured with ICC (2, k) was 0.75 for both the ratings of verbal (*M* = 1.90, *SD* = 0.59) and figural (*M* = 1.95, *SD* = 0.62) responses. We estimated the creative potential of a person (i.e., between-person performance level) via two separate multilevel null models with person as random factor predicting creative ideation performance in the verbal and figural ABC. The resulting intercepts (per person) were used as estimates of each person’s creative potential. This multilevel approach to estimate the creative potential takes the structure of data into account and is largely equivalent to an aggregated mean score per person (see Rominger et al., in pressb).

#### Assessment of contextual information: Consumption of alcohol, caffeine, and nicotine

At each prompt, participants were asked to answer if they had consumed alcohol (within the last 60 min, yes/no), caffeine (within the last 10 min, yes/no), or nicotine (within the last 10 min, yes/no).

#### Between-person criterion variables

##### Creative potential

In order to assess interindividual differences in creative potential, we administered the Test for Creative Thinking—Drawing Production online (TCT-DP; Urban and Jellen, 1995). We asked participants to complete the abstract picture fragments with their image editing software, in a free-associative, and original way. The time limit was 15 min and was monitored during the online meeting. The generated drawings were then sent to the experimenter. Two independent and trained raters (one man and one woman) scored the TCT-DP in accordance with the test manual (e.g., unconventionality, inclusion of new elements, graphic combinations, etc.). Each criterion was scored between 0 and 6. The two scorings showed substantial correlation indicating high interrater reliability (*r* = 0.97). The mean score of both raters per criterion was used as index of creative potential assessed and served as criterion for validity analyses (*M* = 1.56, *SD* = 0.54).

##### Self-assessment of creative ideation behavior

Creative ideation behavior was assessed by a German version of Runco’s Ideational Behavior Scale (RIBS; Runco et al., 2001; see e.g., Diedrich et al., 2018), which includes 17 statements such as “I come up with an idea or solution other people have never thought of.” Participants responded to the items on a scale ranging from 1 (never) to 5 (very often; *M* = 64.59, *SD* = 14.80; α = 0.92). The scale reflects creative ideation skills and is commonly used as a criterion measure of divergent thinking performance (Plucker et al., 2006; Diedrich et al., 2018; for an overview see, Runco et al., 2014).

##### Self-assessment of real-life creativity: Creative activities and creative achievements

As further criterion variables, creative activities (Cact) and creative achievements (Cach) were assessed by means of the Inventory of Creative Activities and Achievements (ICAA; Diedrich et al., 2018). This questionnaire asks for eight different domains of creative activities and achievements (i.e., literature, music, arts and crafts, cooking, sports, visual arts, performing arts, science, and engineering). The sum score of creative activities showed an internal consistency of α = 0.81 and served as an index of everyday little-c creativity. Furthermore, the internal consistency of the sum of creative achievements score was α = 0.70, which indexes Pro-C creativity.

##### Personality assessment

The 60-items NEO-FFI by Costa and McCrae (1992, German translation; Borkenau and Ostendorf, 1997) was used to assess participants’ openness, neuroticism, extraversion, agreeableness, and conscientiousness. Openness in particular was consistently associated with creative ideation performance and the potential for open problem solving (McCrae, 1987; Silvia, 2008). The internal consistency of openness in the present study was α = 0.75 (*M* = 34.48, *SD* = 6.51; neuroticism: α = 0.85, *M* = 22.45, *SD* = 4.39; conscientiousness: α = 0.82, *M* = 31.65, *SD* = 6.96; agreeableness: α = 0.68, *M* = 33.80, *SD* = 5.57; and extraversion: α = 0.73, *M* = 27.39, *SD* = 6.12).

#### Psychometric analysis strategy

##### Reliability analyses

Consistent with Rominger et al. (in pressb), we firstly calculated reliability analyses at the between- (*R*_*KR*_) and the within-person (*R_C*) level for both versions of the ABC separately using generalizability theory analysis (Cronbach, 1972; Brennan, 2001, for review see, Shrout and Lane, 2012). Following Nezlek (2017), we differentiated between Level 1, which were the four raters, Level 2, which were the prompts, and the person level (Level 3). Raters were considered as items (Level 1; Nezlek, 2017) measuring creative ideation performance in different situations in daily life context. Generalizability theory analysis is especially suited to assess reliability of ecological momentary assessment data, allowing the partitioning of between-person, within-person, and error variance by decomposing the observed variance associated with person, item (i.e., rater), measurement occasion (i.e., prompt), and their respective interactions. To estimate reliability, we used the methods described by Revelle and Wilt (2019) by applying the software psych (version 2.2.5; Revelle, 2022) running in R (version 4.2.0; R Core Team, 2021). Secondly, to estimate the stability of the assessed creative potential, creative potential based on the first half of prompts was correlated with the creative potential based on the second half of prompts (i.e., split-half reliability). All reliability analyses were calculated separately for the verbal and figural ABC and corrected with the Spearman-Brown correction formular (Bühner, 2011).

##### Validity analyses

For criterion validity estimates at the between-person level, creative ideation performance in the ABC was correlated with the creative potential assessed by means of the TCT-DP, the self-reported creative behavior, the personality trait openness to experience (as well as the other personality measures for discriminant validity), and the creative activities as well as the creative achievements.

To evaluate the within-person validity, two multi-level models with response time at Level 2 (between-person, group mean) and Level 1 (within-person, group mean centered) and subjective ratings of the creative quality of ideas at Level 2 (between-person, group mean) and Level1 (within-person, group mean centered) as well as gender and age as fixed effects and participants as random effects were calculated. We predicted the (externally rated) creative abilities for the verbal and figural ABC, respectively. Finally, we evaluated meaningful within-person variation of creative abilities associated with the consumption of alcohol, nicotine, and caffein in two further multi-level models.

## Results

### Descriptive statistics

Overall, the ABC delivered 4,600 prompts, of which 3,118 were answered (67.98%). For the verbal ABC, we collected 1,558 ecological momentary assessment responses and for the figural ABC 1,569 ecological momentary assessment responses. Furthermore, 12.75% (*n* = 200) of all completed figural ABC responses got lost randomly due to transmission errors of the drawings. This resulted in a total of 1,549 available ecological momentary assessment responses for the verbal ABC and 1,369 responses for the figural ABC. Incomplete responses were discarded from multi-level analyses (9 responses of the verbal ABC and 1 response of the figural ABC). Alcohol consumption was reported for 61 figural and 72 verbal prompts, nicotine consumption for 60 figural and 65 verbal prompts, and caffeine intake for 80 figural and 98 for verbal prompts.

### Reliability analysis

The between-person variance of creative abilities was between 6 and 7%. The reliabilities of these scores were estimated with *R*_*KR*_ = 0.80 for the verbal and *R*_*KR*_ = 0.85 for the figural ABC. The proportion of within-person variance was 36 and 30%, respectively, with reliability estimates of *R_C* = 0.73 for the verbal and *R_C* = 0.72 for the figural ABC (see Table 1). This indicates that both the verbal and the figural ABC could reliably assess systematic within-person and between-person variations of creative abilities.

**TABLE 1:** Variance decomposition of creative abilities in the verbal and figural ABC task and summary of reliability estimates.

	Verbal ABC task		Figural ABC task	
**Variance component**				
$\sigma_{T\,o\,t\,a\,l}^{2}$	0.63		0.80	
$\sigma_{P}^{2}$	0.04	6.13%	0.06	7.09%
$\sigma_{T}^{2}$	0.00	0.00%	0.00	0.24%
$\sigma_{R}^{2}$	0.03	4.07%	0.13	16.16%
$\sigma_{P\times T}^{2}$	0.23	36.19%	0.24	29.58%
$\sigma_{P\times R}^{2}$	0.01	1.06%	0.01	1.58%
$\sigma_{T\times R}^{2}$	0.00	0.00%	0.00	0.21%
$\sigma_{R\,e\,s\,i\,d\,u\,a\,l\,s}^{2}$	0.33	52.55%	0.36	45.14%
**GT reliability estimates**				
*R*_*KR*_ (*R*_*KRn*_)	0.80 (0.80)		0.85 (0.85)	
*R_C* (*R*_*Cn*_)	0.73 (0.71)		0.72 (0.62)	

σ^2^, variance component; P, person; T, time; R, rater; R_KR_, between-person reliability; R_KRn_, nested between-person reliability; R_C_, within-person reliability; R_Cn_, nested within-person reliability.

### Stability

**FIGURE 1:**
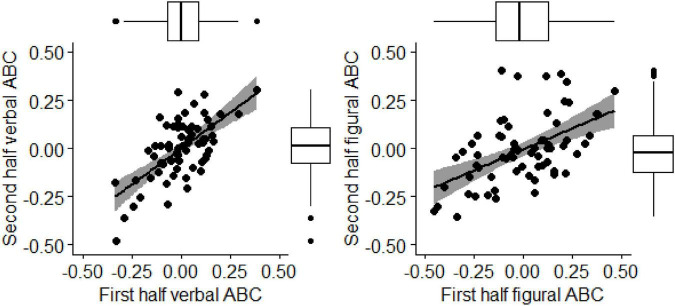
Scatter plot of first and second half performance (i.e., split-half stability) for the verbal **(left)** and the figural ABC **(right)**. Slope and corresponding credibility intervals are depicted.

In addition to reliability estimates, we explored the stability of the between-person creative ability scores for the verbal and the figural ABC. The first half of prompts was correlated with performance estimation based on the second half of prompts. This analysis showed evidence for good split-half reliability of the verbal ABC (*r* = 0.65; Spearman-Brown corrected *r* = 0.79) and the figural ABC (*r* = 0.57; Spearman-Brown corrected *r* = 0.73, see Figure 1). These analyses indicate that the verbal and the figural ABC delivers reliable and stable estimates of creative potential.

### Evidence for between-person validity

#### Creative potential

The creative ideation performance on the verbal ABC significantly correlated with the creative potential assessed by means of the TCT-DP (*r* = 0.27, *p* = 0.024). Similarly, the figural ABC also positively correlated with the TCT-DP (*r* = 0.31, *p* = 0.010). This indicates criterion validity of both the verbal and the figural ABC, which was further strengthened by a significant correlation between both versions of the ABC (*r* = 0.52, *p* < 0.001).

#### Self-assessment of creative ideation behavior, creative activities, and creative achievements

The self-reported creative ideation behavior (RIBS) was significantly associated with the verbal ABC (*r* = 0.31, *p* = 0.009). The between-person level of the performance in the verbal ABC was also associated with creative achievements (Cach: *r* = 0.31, *p* = 0.010) and creative activities (Cact: *r* = 0.32, *p* = 0.008) of the ICAA. The performance assessed by means of the figural ABC was not correlated with these measures (see Table 2).

**TABLE 2:** Overview of Pearson correlations of between-person variables, separately for the verbal and the figural ABC.

	Verbal ABC (*p*)	Figural ABC (*p*)
Creative potential (TCT-DP)	0.27 (0.024)	0.31 (0.010)
Creative ideation behavior (RIBS)	0.31 (0.010)	−0.00 (0.995)
Creative activities (Cact, ICAA)	0.32 (0.008)	−0.09 (0.476)
Creative achievements (Cach, ICAA)	0.31 (0.010)	−0.12 (0.322)
Openness (NEO-FFI)	0.43 (<0.001)	0.09 (0.442)
Neuroticism (NEO-FFI)	−0.11 (0.371)	−0.14 (0.247)
Conscientiousness (NEO-FFI)	0.15 (0.208)	0.11 (0.360)
Agreeableness (NEO-FFI)	−0.06 (0.653)	0.12 (0.323)
Extraversion (NEO-FFI)	−0.06 (0.618)	−0.22 (0.064)
Figural ABC	0.52 (<0.001)	

#### Personality

The personality trait openness to experience was significantly correlated with the creative potential assessed by the verbal ABC (*r* = 0.43, *p* < 0.001). Neuroticism, conscientiousness, agreeableness, and extraversion showed no significant association with creative performance, which points to evidence of discriminant validity (see Table 2). The between-person variation of the figural ABC performance was not significantly associated with any measure of the Big-Five (see Table 2). However, extraversion showed a trend for a negative association with the figural ABC (*r* = −0.22, *p* = 0.064).

#### Evidence for within-person validity of the ambulatory battery of creativity

##### Associations of response time and subjective originality ratings with verbal and figural ambulatory battery of creativity performance

The multilevel model for the verbal ABC showed that higher average response times, but not the subjective originality ratings (at the person Level 2) predicted creative abilities (see Table 3). On the within-person level (Level 1, group mean centered), longer response times (at a trend level) and higher subjective creativity ratings were associated with higher creative ideation performance (see Table 3).

**TABLE 3:** Multilevel model predicting verbal ABC abilities.

Parameter	Estimate (SE)	*df*	*t*	*p*
*Intercept*	1.68 (0.17)	1,478	9.63	<0.001
**Level 2**				
Age	0.00 (0.01)	64	0.38	0.703
Sex (1 = women, 0 = men)	−0.08 (0.06)	64	−1.47	0.146
Subjective originality rating	0.11 (0.24)	64	0.47	0.638
Response time	0.75 (0.24)	64	3.08	0.003
**Level 1 (group mean centered)**				
Subjective originality ratings	0.90 (0.07)	1,478	13.37	<0.001
Response time (creative ideation)	0.12 (0.07)	1,478	1.80	0.072

Subjective originality ratings transformed to range between 0 and 1, response times are in minutes.

The pattern of findings at Level 1 was virtually the same for the figural ABC (see Table 4). All three group mean centered variables (i.e., subjective originality ratings, response time, and drawing time) significantly predicted the creative performance outcome of the figural ABC (see Table 4). Furthermore, subjective ratings at Level 2 as well as the drawing time at Level 2 also predicted originality of the figural ABC, but not the group mean of the response time.

**TABLE 4:** Multilevel model predicting figural ABC abilities.

Parameter	Estimate (SE)	*df*	*t*	*p*
*Intercept*	1.00 (0.20)	1,296	4.86	<0.001
**Level 2**				
Age	0.01 (0.01)	63	1.95	0.056
Sex (1 = women, 0 = men)	−0.04 (0.06)	63	−0.71	0.478
Subjective originality rating	0.68 (0.23)	63	3.03	0.004
Response time (creative ideation)	0.45 (0.46)	63	0.96	0.338
Drawing time (idea elaboration)	0.67 (0.19)	63	3.44	0.001
**Level 1 (group mean centered)**				
Subjective originality rating	0.98 (0.07)	1,296	13.80	<0.001
Response time (creative ideation)	0.68 (0.12)	1,296	5.42	<0.001
Drawing time (idea elaboration)	0.46 (0.07)	1,296	6.46	<0.001

Subjective originality ratings transformed to range between 0 and 1, response times are in minutes.

##### Contextual co-variation of creative abilities in the verbal and figural ambulatory battery of creativity as an additional indicator of within-person validity

As a further evaluation of the within-person validity of the ABC, we investigated if the consumption of alcohol, caffeine, and nicotine is associated with creative ideation performance in everyday life. For the verbal ABC, alcohol consumption significantly predicted creative performance of the associated prompt (see Table 5).

**TABLE 5:** Multilevel model for the consumption of alcohol, caffeine, and nicotine predicting performance in the verbal and the figural ABC.

Parameter	Estimate (SE)	*df*	*t*	*p*
Verbal ABC				
*Intercept*	1.90 (0.03)	1,476	65.85	<0.001
Nicotine	−0.12 (0.08)	1,476	−1.51	0.132
Alcohol	0.15 (0.08)	1,476	2.09	0.037
Caffeine	0.01 (0.06)	1,476	0.21	0.833
Figural ABC				
*Intercept*	1.96 (0.03)	1,294	57.73	<0.001
Nicotine	−0.19 (0.09)	1,294	−2.09	0.037
Alcohol	−0.16 (0.08)	1,294	−1.99	0.047
Caffeine	0.13 (0.07)	1,294	1.83	0.067

Nicotine and alcohol consumption negatively predicted the figural creative ability of the associated prompt. Furthermore, caffeine consumption positively predicted creative ideation performance of the figural ABC at a trend level (see Table 5).

## Discussion

This study aimed to replicate and extend previous findings on the psychometric properties of the novel ABC, which assesses creative ideation performance multiple times in the field. Thus, the ABC seeks to measure people’s creative potential as well as the moment-to-moment fluctuations of creative abilities (Rominger et al., in pressa). We replicated the promising reliability estimates shown previously (Rominger et al., in pressb) and observed additional evidence for criterion validity, which suggests that the ABC is well suited to assess interindividual differences (i.e., people’s creative potential, between-person variance) as well as moment-to-moment fluctuations of creative abilities (i.e., within-person variance) in everyday life contexts. Therefore, this instrument, designed to capture dynamic variation as well as static individual differences in creative abilities, can be applied to address new questions in creativity research from a new, fresh, and ecologically more valid perspective (Rominger et al., in pressa).

The present application of the ABC replicates the proportion of variance reported by Rominger et al. (in pressb), where creative abilities showed a higher variation within person (∼33%) compared to between person (∼8%). This division of variances underscores the importance to assess creative ideation performance multiple times within an ever-changing environment, or otherwise creativity research would miss a meaningful source of information.

### Should we apply the ambulatory battery of creativity to assess a person’s creative potential?

Despite the circumstance that the present findings are based on a young academic sample of moderate size, at least for the verbal ABC the answer is a clear yes. Here, taking the effort to assess a person’s creative potential throughout diverse situations of everyday life provides an ecologically valid estimate of people’s creative abilities as exhibited outside the laboratory and in everyday life. Although the proportion of variance at the level of a person was only 6%, the reliability estimate was good, which was further strengthened by a good stability of the estimated creative potential. Replicating the findings of Rominger et al. (in pressb), the between-person level of creative performance showed the expected associations with a laboratory measure of creative potential (TCT-DP), self-reported creative behavior (RIBS), and the personality trait openness (McCrae, 1987; Silvia, 2008). Given the reliability and validity evidence of the verbal ABC, this instrument constitutes an important extension of psychometrically sound methods to study creative abilities in the field. Additionally, the present study reported an association of the verbal ABC with creative activities and creative achievements (Jauk et al., 2014; Diedrich et al., 2018), which indicates that the verbal version of the ABC captures individual differences in creative potential predicting aspects of Little-c and Pro-c creativity.

Although, both measures of the ABC were substantially correlated with each other (*r* = 0.52) and with the TCT-DP, the figural ABC showed a divergent correlation pattern. It may be assumed that the verbal and the figural ABC might assess different aspects of creativity (at least at the level of a person). In contrast to the verbal version, the figural ABC might more strongly capture aspects of mini-c creativity (i.e., personally meaningful creative experiences; Kaufman and Beghetto, 2009). In line with this assumption, we found no association of the figural ABC with creative achievements and creative activities. However, people who considered their drawings as more original (at the aggregated level of a person, Level 2) showed a higher creative potential in the figural domain. Additionally, although most available research indicated virtually no or at best only small positive associations between extraversion and creative potential measures (Fink and Neubauer, 2008; Kandler et al., 2016; Puryear et al., 2017), the figural ABC was negatively associated with extraversion by trend. Since this constitutes a novel finding by means of the ambulatory assessment of figural creativity, future studies should investigate its robustness and replicability.

Taken together, the ABC constitutes a promising instrument to capture interindividual differences of creative potential in the verbal and the figural domain. The additional effort, in contrast to assessing creative ideation performance only once (or twice; Erwin et al., 2022), is justified by the benefit of a measure with a higher ecological validity, which might reveal associations that are not observed when creative abilities are assessed under more constant conditions (Rominger et al., in pressa).

### Should we use the ambulatory battery of creativity to assess within-person variation of creative abilities?

Our recommendation is yes. As stated by Rominger et al. (in pressa), beside the increased ecological validity, the assessment of within-person variation and the dynamic fluctuation of creative abilities constitutes a promising advantage of the ABC in the field. Measuring creative ideation performance only once would overlook the meaningful distribution of variation of creative abilities in people’s everyday life.

Most of the psychometric properties of the verbal and figural ABC are comparable. We found a considerable amount of variation within person (∼30%; Dirk and Schmiedek, 2016; Sliwinski et al., 2018), which crucially justifies the additional effort of studying fluctuations of creative abilities multiple times in ever changing environments of people’s daily lives. Furthermore, the reliability estimates were high for both ABC versions (≥0.70), especially when comparing the reliability estimates with cognitive tasks assessed in the field (see e.g., Sliwinski et al., 2018). This difference in reliability estimates between cognitive and creative ideation measures might be due to the nature of the quantification of the measures itself. High quality of interrater agreement of the originality ratings by external and independent judges is an important prerequisite of the ABC and strongly impact the reliability of the observed within-person variation. In contrast, however, reliability of cognitive performance in ecological momentary assessments is quantified via the number of correct answers (and reaction times) of at least two items presented per prompt.

Furthermore, both, the verbal and the figural ABC measure meaningful variations of creative abilities over time and situations, which were associated with the fluctuation of subjective ratings as well as the response times during the tasks. In accordance with Rominger et al. (in pressb), subjective ratings and response times positively predicted the creative ideation outcome of each prompt. The response times might serve as an indicator of the creative ideation process associated with the effort and the number of generated ideas to finally reach an original idea (i.e., fluency component of creativity; see e.g., Barbot, 2018). Furthermore, the drawing time, as an indicator of idea elaboration, additionally predicted the originality of each drawing (Barbot, 2018).

Analyzing the effects of situational context on creative abilities, we found that prompts associated with alcohol consumption predicted higher performance in the verbal ABC, but less creative outcome in the figural ABC. Alcohol is known to reduce cognitive control, but no consistent associations with creative performance have been observed with conventional assessments so far (Jarosz et al., 2012; Benedek et al., 2017; Benedek and Zöhrer, 2020). This might point to a high sensitivity and a potential domain specificity of the ABC versions with respect to within-person performance variations. Furthermore, nicotine showed a negative association with fluctuation of creative abilities assessed by means of the figural ABC, which constitutes a novel finding since previous studies investigating interindividual differences of tobacco use found no substantial associations with creativity (Plucker and Dana, 1998; Plucker et al., 2009). Additionally, caffeine seemed to positively predict the originality of the figural ABC (Zabelina and Silvia, 2020). This pattern of intraindividual effects indicate validity of the assessed moment-to-moment fluctuation. However, not at least due to the comparatively low number of prompts associated with substance consumption in the present study (e.g., ∼60 for nicotine consumption), these findings need replication in future studies applying the ABC in contexts with a higher probability of alcohol intake and nicotine consumption (e.g., later assessment time, experimental manipulation of substance intake during conducting an ecological momentary assessment protocol). Furthermore, beyond the pure effects of substance use on the creative ideation performance it might also be assumed that other contextual variables such as social context (which might be associated with alcohol and nicotine consumption) might have impacted creative ideation performance in the present study.

## Conclusion and future perspectives

In accordance with the tradition of ambulatory assessment of cognitive performance and intelligence, the reliable and stable assessment of creative abilities may serve as an ecologically valid marker of health and illness (Waters and Li, 2008; Koo and Vizer, 2019; Papp et al., 2021; van Patten et al., 2021; Zlatar et al., 2022). Furthermore, the measurement of creative abilities and creative cognitive functions could offer meaningful, incremental, important, and ecologically more valid information about a person’s state of health as well as state of illness, which could be useful in eHealth/mHealth approaches. Original and useful ideas can help in handling daily problems (Perchtold-Stefan et al., in press), which makes creative ideation crucial for an adaptive interaction with the environment and determines the success of our everyday life functioning. This supports the assumption that creative abilities can serve as an index of brain health and cognitive reserve (e.g., Fusi et al., 2020; Rominger et al., 2022), which is further strengthened by the findings that creative abilities are (multidimensionally) associated with effects of aging (Fusi et al., 2020), wellbeing, positive affect (Rominger et al., 2020a), mindfulness (Baas et al., 2014), as well as physical activity (Rominger et al., 2020a,^2022^). Similar to these variables of health, the ecologically valid measure of creative abilities might also provide relevant information about clinical samples such as patients suffering from schizophrenia (Acar et al., 2018) and (frontotemporal) dementia (Fusi et al., 2021).

The application of the ABC seems especially advantageous when information about the temporal dynamics of creative abilities as well as potential influences of contextual variables are of interest (Weizenbaum et al., 2020) such as tracking creative performance in digital health interventions or person-centered care and assessment concepts (Koo and Vizer, 2019). Knowing in which contexts people show higher creative abilities might allow to derive at personalized advises to reach specific contexts if more creative abilities are needed. These opportunities will greatly expand the avenues for future creativity research topics toward the dynamics of creative thinking abilities and facilitate new insights into the mechanisms of creative cognition in healthy as well as clinically relevant samples (Rominger et al., in pressa).

By applying the ABC, future studies could target the assessment of dynamic changes of creative abilities in people’s everyday life in an ecologically valid manner. This enables a new and fresh perspective on creativity research by focusing on the moment-to-moment fluctuations of creative abilities. Future studies could advance this approach by adding further psychological or contextual variables such as affect, wellbeing, and attention as well as environmental surroundings, participant’s physical activity, or neurophysiological indicators (e.g., ECG, EEG; Weizenbaum et al., 2020), which might allow psychophysiological triggering and robust predictions of creative states (Agnoli et al., 2020; Schwerdtfeger and Rominger, 2021; Rominger and Schwerdtfeger, 2022; Rominger et al., in pressc). A more complex assessment of psychological and psychophysiological states in combination with the ABC could gain further highly relevant information on how, when, and why people show higher creative abilities.

## Data availability statement

The raw data supporting the conclusions of this article will be made available by the authors, without undue reservation.

## Ethics statement

The studies involving human participants were reviewed and approved by Karl-Franzens-Universität Graz Ethikkomission (Universitätsplatz 3, 8010 Graz, Austria). The patients/participants provided their written informed consent to participate in this study.

## Author contributions

CR, AF, and AS contributed to the conception and design of the study. CR and BW organized the database. CR performed the statistical analysis and wrote the first draft of the manuscript. CR, AF, MB, CP-S, and AS wrote sections of the manuscript. All authors contributed to the manuscript revision, read, and approved the submitted version.
